# The Effect of Humidity on the Dissolution Kinetics and Tablet Properties of Immediate-Release Tablet Formulation Containing Lamotrigine

**DOI:** 10.3390/pharmaceutics14102096

**Published:** 2022-09-30

**Authors:** Mladena Lalić-Popović, Gordana Švonja Parezanović, Nemanja Todorović, Zoran Zeković, Branimir Pavlić, Nataša Milošević, Jelena Čanji Panić, Ana Stjepanović, Ljiljana Andrijević

**Affiliations:** 1 Department of Pharmacy, Faculty of Medicine, University of Novi Sad, 21000 Novi Sad, Serbia; 2 Centre for Medical and Pharmaceutical Investigations and Quality Control (CEMPhIC), Faculty of Medicine, University of Novi Sad, 21000 Novi Sad, Serbia; 3 Faculty of Technology Novi Sad, University of Novi Sad, 21000 Novi Sad, Serbia; 4 Department of Biochemistry, Faculty of Medicine, University of Novi Sad, 21000 Novi Sad, Serbia

**Keywords:** lamotrigine, dissolution test, stability testing, experimental design, moisture

## Abstract

This study aims to find the effects of high (75%) and low (30%) humidity conditions and its correlation with formulation composition on dissolution kinetics of lamotrigine (LMT) from prepared immediate-release tablets during one- and four-week periods. Two types of fillers microcrystalline cellulose (MCC) or anhydrous lactose (LAC), disintegrant sodium starch glycolate (NaSG, 0.5% or 4%), and lubricant magnesium stearate (MgST, 0.25% or 5%) were used. A three-factor two-stage complete factorial design (2^3^) was used to assess the influence of the composition of the tested formulations. The tablets were produced by direct compression and characterized using a disintegration test, a resistance to crushing test, and dissolution tests (pH 1.2 and pH 6.8). Using Design Expert software, it was concluded that in addition to the effect of fillers on pH 6.8, NaSG has a significant impact after exposure to high and low humidity, as well as its interaction with LAC and MCC. In the dissolution medium pH 1.2, under conditions of high humidity, the content of MgST and NaSG and their interaction show a significant influence. The release rate of LMT was affected by humidity as well as type of excipients and their interactions.

## 1. Introduction

The successful formulation of stable and effective tablets requires a careful selection of the excipients, since fluctuations in the properties of the excipients have a great influence on the stability, release rate, and kinetics. In order to achieve the targeted therapeutic effectiveness and safety of tablets, it is necessary to know the effect of auxiliary substances on the stability of the drug, which has a direct effect on the stability of the drug [1,2,3]. The rate of dissolution/release and the permeability of the drug from tablet formulations are the fundamental parameters that control the rate and degree of absorption of the drug, while the influence of the components of the formulation is most pronounced in tablets with a narrow therapeutic index and poor solubility of active pharmaceutical ingredient (API) [4].

Humidity is an interesting factor that could often not be neglected because patients use dose administration aids (DAAs) especially during chronic therapy, where daily, weekly, or monthly therapy can be separated. DAAs, due to the lack of hermeticity, offers varying levels of protection from humidity to repackaged medicine [5,6,7]. During this manipulation, dosage forms are removed from the original package, a factor not taken into account in a standard stability study, which usually lasts six months. During stability studies of solid dosage forms, apart from organoleptic charcteristics, mechanical characteristics are also monitored. For drugs with poor solubility special attention is given to dissolution profile changes. Changes in mechanical characteristics and dissolution rates for some medicines could lead to deprivated therapeutic efficacy. Problems with repacking medicine into DAAs is that tablets are exposed to fluctuations in humidity depending on the climate, season, and storage conditions, which calls into question the effectiveness of the therapy, and it is not foreseen in standard stability studies.

Antiepileptics are an example of a group of drugs in which a small change in the dissolution/release rate of API can affect the therapeutic efficiency, which could lead to recurrence of epileptic seizure or cause side effects. Namely, the ideal antiepileptic is the one that can prevent convulsive attacks and at the same time has no unwanted effects. The problem with the formulation of antiepileptics becomes more difficult if it is a poorly soluble active pharmaceutical ingredients (API), such as lamotrigine (LMT), when the influence of excipients from the tablet and the influence of storage factors on therapeutic efficiency come into focus, which is why finding the optimal formulation of these drugs is very complicated. LMT belongs to class II according to the Biopharmaceutical Bisystem of Classification (BSC) [8], which is characterized by good permeability and poor solubility in water, and the rate of dissolution/release of LMT is a limiting factor that affects the rate of absorption and its biological availability. Based on the above, a major problem in the development of formulations of LMT tablets is the choice and optimal proportion of excipients, the slightest variation of which can cause therapeutic failure or cause an unwanted reaction.

The characterization of the tablet material and the connection of the critical characteristics of the tablet mixture with the physical and mechanical characteristics of the tablet is one of the tasks necessary to formulate an optimal tablet [9]. Examining the physical properties of the tablet material makes it possible to predict the influence of the powder characteristics on the critical characteristics of the tablet as the final form of the drug [10]. One of the limiting factors at the beginning of tablet formulation development is the limited amount and high cost of API and excipients that are available to perform the necessary experiments. Therefore, application of experimental design (DoE) as a method of setting up experiments and statistical measurement of the influence of certain factors significantly contributes to the pharmaceutical industry by shortening the analysis time. The choice of appropriate excipients in tablets, such as the amount and ratio of binding agent, disintegrating agent and sliding agent, is one of the key parameters of the formulation in the tablet production phase in order to obtain appropriate API dissolution kinetics [11]. After a successfully formulated tablet, the method of storing both the raw materials and the finished tablets is important, which depending on the different humidity conditions can significantly affect the characteristics of the tablets and thus change the release of API.

Determination of formulation robustness to humidity is important if medicine is expected to be repackaged into DAAs. Thus, the aim of this study was to evaluate the effects of increased and decreased humidity (i.e., 30% and 75% relative humidity) on prepared LMT immediate-release tablets over a period of four weeks. Additionally, through application of DoE influence and its mutual interaction is examined to determine to what extent humidity and selected excipients affects the release of LMT.

## 2. Materials and Methods

### 2.1. Materials

Lamotrigine [6-(2,3-dichlorophenyl)-1,2,4-triazine-3,5-diamine] (Jubilant Generics Limited, Uttar Pradesh, India; donated by Alkaloid, Skopje, North Macedonia) is used in the work as a pharmaceutical active substance for the preparation of the tablet formulation. In addition to the active pharmaceutical substance LMT, the following excipients are used in the preparations: anhydrous lactose (Super Tab 21AN, DFE Pharma, Goch, Germany, donated by Galenika, Belgrade, Serbia); microcrystalline cellulose (Vivapur^®^101, JRS Pharma, Rosenberg, Germany); magnesium stearate (Magnesium stearate, Mosselman, Germany); sodium starch glycolate (Primojel^®^, DFE Pharma, Goch, Germany; donated by Galenika, Belgrade, Serbia).

The dissolution medium pH 1.2 (hydrochloric acid, 0.1 M) was prepared using concentrated hydrochloric acid (37%, PanReac, p.a., Barcelona, Spain) and potassium chloride (Lachner, p.a., Neratovice, Czech Republic). Medium pH 6.8 (phosphate buffer, 0.1 M) was prepared using potassium dihydrogen phosphate (Lachner, p.a., Neratovice, Czech Republic) and sodium hydroxide (PanReac, p.a., Barcelona, Spain). Dissolution medias were prepared using United State Pharmacopeia 44-NF 39 [12].

Sodium chloride (JTBaker, p.a., Phillipsburg, NJ, USA) and magnesium chloride (JTBaker, p.a., Phillipsburg, NJ, USA) were used to prepare the solution for adjusting the conditions of increased and decreased humidity.

### 2.2. Experimental Design 

Powder formulations were prepared by mixing different concentration of MgSt (0.25% and 5%) and SSG (0.5% and 4%) with microcrystalline cellulose (MCC) (Vivapur^®^101) or spray-dried lactose (LAC) (Super Tab 21AN) as diluents. A dose of lamotrigine (LMT) was aimed to be 25 mg in each tablet and MCC and LAC amount were adjusted accordingly in every formulation considering that resistance to crushing of tablets should be between 40–60 N. 

Eight powder formulations were prepared using as filler LAC and MCC, SSG as superdesintegrator, and MgSt as lubricant in ratios as follows in Table 1. 

To assess the influence of the composition of the tested formulations, a complete 2^3^ factor at two levels of design was applied, monitoring three factors as components of the formulation, on both parts. 

Experimental runs were designed by Design Expert 10.0.1 (version 8.0.4; Stat-Ease, Inc., Minneapolis, MN, USA) software following full factorial method. 2^3^ full factorial design was applied for examining three variables (factors) at two levels with a minimum of 8 runs. The variables screened for the study were type of binders (X_1_), amount of SSG (X_2_), and amount of MgSt (X_3_) (Table 1). 

### 2.3. Preparation of Tablets

A total of 16 tablet formulations were made: eight placebo formulations F1-F8 (formulations having only excipients, without the medicinal component lamotrigine) and eight formulations with LMT T1-T8. Excipients included lubricants, disintegrants, and binders. Formulations were prepared as per Design of Experiment (DoE) runs. All the ingredients except magnesium stearate were weighed and powder was mixed in powder mixer (Farmalabore tech, Canosa di Puglia, Italy) for 5 min. Then, MgSt was added to the formulation and mixed for 2 min. The tablets were prepared by direct compression using a single punch tableting machine (Korsch EK3, Berlin, Germany).

### 2.4. Setting Conditions for High (75%) and Low (30%) Humidity

All tablet samples were exposed to conditions of increased (75 ± 5%) and decreased (30 ± 5%) humidity, at room temperature (25 ± 2 °C). Tablet samples were selected depending on the formulation. Conditions of increased humidity were achieved in a closed system (desiccator) using saturated aqueous sodium chloride solution (JTBaker, p.a.). Conditions of reduced humidity were achieved in a closed system (desiccator) using saturated aqueous solution of magnesium chloride at room temperature (25 °C) according to previously published methods [13,14]. Conditions in the desiccator were monitored by a thermo-hygrometer (PCE-HT 72-ICA, PCE Instruments, Southampton, UK).

According to the World health organization (WHO), there are four climatic zones. In the document Stability Testing of Active Pharmaceutical Ingredients and Finished Pharmaceutical Products, suggested temperatures are 25 °C or 30 °C, with a relative humidity of 50–75% [15]. Decreased humidity conditions are not suggested in this document. However, the ICHQA1 (R2) document suggests investigations according to climatic zones [16]. Lower humidity in long term stability studies is 35%; in this study was lower humidity was adjusted at 30%. Generally, humidity conditions were chosen based on the study Maclean et al., where the role of excipients in the physical stability of tablets was investigated after being exposed to humidity of 30% and 75% [17]. A research protocol was created to uniquely mimic most possible conditions after repackaging the drug into dose administration aids (DAAs) in continental Europe. Hermecity is a problem with DAAs, thus temperature was standard room temperature (25 °C) and humidity conditions varied.

### 2.5. Evaluation of Flow Properties of Powder Blend

The parameters that were measured to evaluate the flow properties are angle of repose, bulk density, and tapped density according to the suitable monograph in the 10th European pharmacopoeia (Ph. Eur. 10) [18].

For determination of the angle of repose, a fixed funnel method was used. Angle of repose is related to interparticle frictions, and flow properties were determined using the table in chapter 2.9.36. of Ph. Eur. 10 [18]. The powder blend is poured through a funnel to form a cone. The height of the resulting cone (h) is divided by half the width of the base of the cone (r). The inverse tangent of this ratio is defined as the angle of repose (Equation (1)).

(1) $$\theta =tan^{-1}\left(\frac{h}{r}\right)$$

Bulk/tapped volume and density were determined using a tap density powder tester (Erweka typ GTB, Langen, Germany) as required in chapter 2.9.36 in Ph. Eur. 10 [18]. A weighed amount (50 g) of powder blend was filled into graduated cylinder of the apparatus. Unsettled (bulk) volume V0 was read and noted. The cylinder was tapped 10, 500 and 1250 times and corresponding volumes V10, V500, and V1250 were noted. The measurements were taken in triplicate, and each result was expressed as mean and the standard deviation. Bulk and tapped (settled) densities of the blend were calculated as follows Equations (2) and (3):

(2) $$\mathrm{Bulk}\;\mathrm{density}=\frac{m}{Vo}$$

(3) $$\mathrm{Tapped}\;\mathrm{density}=\frac{m}{V}$$

where m is the weight of the sample (g), *Vo* is the bulk volume (mL), and V1250 (mL) is the tapped (settled) volume recorded after 1250. A compressibility index (CI) of the powder mixtures was calculated using Equation (4) and the Hausner ratio (HR) of powder mixtures was calculated using Equation (5):

(4) $$\mathrm{CI}=\frac{Vo}{Vf}\times 100$$

(5) $$\mathrm{HR}=\frac{Vo}{Vf}$$

where *Vf* is the final tapped volume obtained for the powder mixtures. 

### 2.6. Characteristics of Tablets Evaluation

All prepared tablet formulations (placebo and with LMT) were evaluated for resistance to crushing, weight variation, thickness, friability, disintegration, and dissolution characteristics.

Resistance to crushing of tablets was tested according to the requirements of Ph. Eur.10, chapter 2.9.8 [18], using diametrical pressure (Erweka TBH 28, Langen, Germany). Measurements were performed with ten samples each. The measurement results are expressed as the mean of all the measured forces required to break the tablets. All results are expressed in newtons (N).

The tablet disintegration test was performed according to the requirements of Ph. Eur. 10, chapter 2.9.1. [18]. Disintegration was measured using a standard device for determining disintegration (Erweka ZT54, Langen, Germany). The disintegration test was performed with six samples of tablets. The tablets were placed individually in each cylinder and the cylinder holder was immersed in a beaker of medium. Distilled water heated to 37 ± 0.5 °C was used as the test medium. The disintegration of the tablets was visually monitored, and the end of the test was calculated as the moment when the last tablet of six samples disintegrated (perished through a sieve at the bottom of the cylinder). According to the request of Ph. Eur. 10 uncoated immediate release tablets should disintegrate within 15 min [18].

The friability test was performed according to the requirements of Ph. Eur. 10, chapter 2.9.7. [18]. A total of 20 tablets were selected from each batch and weighed. Each group of tablets was rotated at 25 rpm for 4 min (100 rotations) using friability tester (Roche friabilator). The tablets were then dusted and re-weighed to determine the loss in weight. Friability was then calculated as percent weight loss from a mass of 20 tablets before friability test, and tablet formulation was considered acceptable if less than 1% of their weight were lost.

Uniformity of content test was performed with 10 tablets according to chapter 2.9.6. in Ph. Eur. 10 from each series. Tablets were taken at random and powdered, then dissolved in pH 6.8 dissolution medium and concentration was measured using UV/Vis spectrometry method. According to the Ph. Eur. 10 criteria in test A, each individual content should be between 85% and 115% of the average content value [18].

The rate of dissolution/release of LMT from various formulations was tested using apparatus 2 (paddle apparatus) according to chapter 2.9.3 in Ph. Eur 10 at a speed of 50 rpm [18], on an Erweka model DT800 (Erweka, Langen (Hessen), Germany).

Solutions of two pH values, prepared according to the requirements of the United State Pharmacopeia 44-NF 39 [12], hydrochloric acid solution pH 1.2 and phosphate buffer pH 6.8, at a temperature of 37 ± 0.5 °C were used as medium. A total of 900 mL of dissolution medium was used. Six samples of tablets were used to test the dissolution rate. The dissolution rate of LMT was examined for 30 min in pH 1.2 and 60 min in pH 6.8. Dissolution time at pH 1.2 was adjusted according to Food and Drug Administration (FDA) Dissolution Methods Database where are recommended dissolution methods for drug products. For LMT tablets with immediate release recommended dissolution testing is in medium pH 1.2 for 30 min. The above mentioned tablet testing was performed before and after exposure to humidity condition of increased (75 ± 5%) and decreased (30 ± 5%) humidity, at room temperature (25 ± 2 °C). All formulations are held in altered humidity conditions for four weeks.

### 2.7. UV/Vis Spectrophotometry

The content of LMT in tablets and in dissolution samples was determined by measuring the absorbance using UV/Vis spectrophotometric method, which was previously published and confirmed [19,20]. The absorbance of LMT was measured at a wavelength of 267 nm in pH 1.2 dissolution medium [19] and 304 nm in pH 6.8 dissolution medium [20].

### 2.8. Statistical Analysis

To confirm the half-normal plots, ANOVA tests and Pareto charts were generated using Design Expert^®^ (version 8.0.4; Stat-Ease, Inc., Minneapolis, MN, USA); a significant threshold of *p* < 0.05 was used. The Pareto charts help to visualize the relative size of each effect.

## 3. Results

### 3.1. Powder Blend Flowability

The flowability of individual excipients, LMT, as well as a mixture of powder formulations was tested. By examining the value of bulk density, it was determined that the lowest bulk density has MgST (0.28 g/cm^3^), LMT (0.29 g/cm^3^), then MCC (0.37 g/cm^3^), and the highest density has LAC (0. 71 g/cm^3^) and NaSG (0.79 g/ cm^3^). 

The angle of repose of the used excipients showed that the smallest angle of repose has MgST (27.4°) and the highest NaSG (41.32°). Of the replenishments, LAC had a 36.66° angle of repose, while MCC had a better angle of repose 28.4°, although both belong to the group with good flow properties according to Ph. Eur. 10 [15]. LMT was the only excipient with an angle of repose of 42.86° and thus showed a poor flowability according to both tests. 

Based on HR and CI values, MCC and MgST have good flowability (MCC HR-1.32 and CI-24.07% MgST HR-1.23 and CI-19.23%), while LAC and NaSG have a poor flowability (LAC HR-1.36 and CI-26.67%, NaSG HR-1.39 and CI-28.33%) and LMT an extremely poor flowability (LMT HR-1.75 and CI-42.86%). 

**Figure 1:**
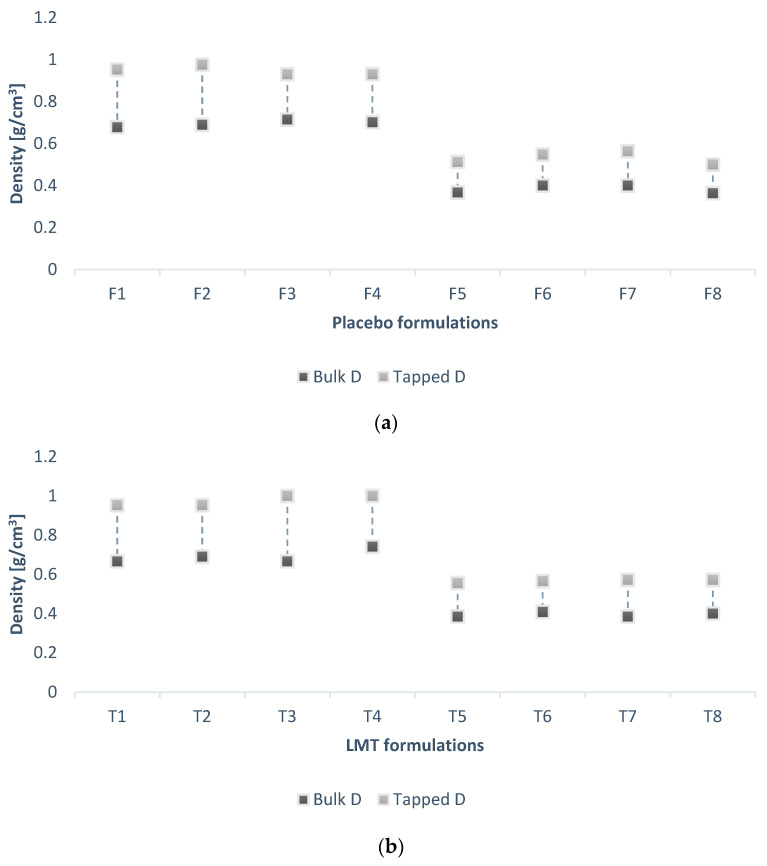
Comparative diagram of bulk and tapped densities of (**a**) placebo formulations (F1–F8) and (**b**) formulations with LMT (T1–T8).

Each tablet contains 25 mg of LMT as API. Due to the difference in the density of the filler and the weight of the tablets, the concentration of LMT was 5% in formulations with LAC and 7% in formulations with MCC. Bulk density of formulations with LMT (T1-T8) is in the range of 0.38–0.74 g/cm^3^; tapped density between 0.56 and 1 g/cm^3^, CI 25.9–32.69%, or HR from 1.35 to 1.5. The comparative diagram (Figure 1) of bulk and tapped densities of formulations with LMT indicates the largest difference in the formulation of T3 and therefore the largest CI. 

Observing the comparison diagram (Figure 1) for bulk and tapped density, the difference between the formulations can be noticed in placebo and LMT formulations. The CI formulation with LAC (T1-T4) ranges from 23.2% to 29.3% or HR from 1.30 to 1.41, while in formulations with MCC (T5-T8) CI has a value of 26.0% to 28.4%, and HR from 1.35 to 1.40. Formulation F2 had the largest difference between bulk and tapped density, with the highest CI of 29.3%. Formulation F2 has high proportions of MgST and NaSG, so it must be emphasized that due to the high content of MgST, such poor flow rate of this formulation is not expected. However, this parameter only serves as a guideline for estimating powder flow and must be combined with other methods, such as angle of repose. 

**Figure 2:**
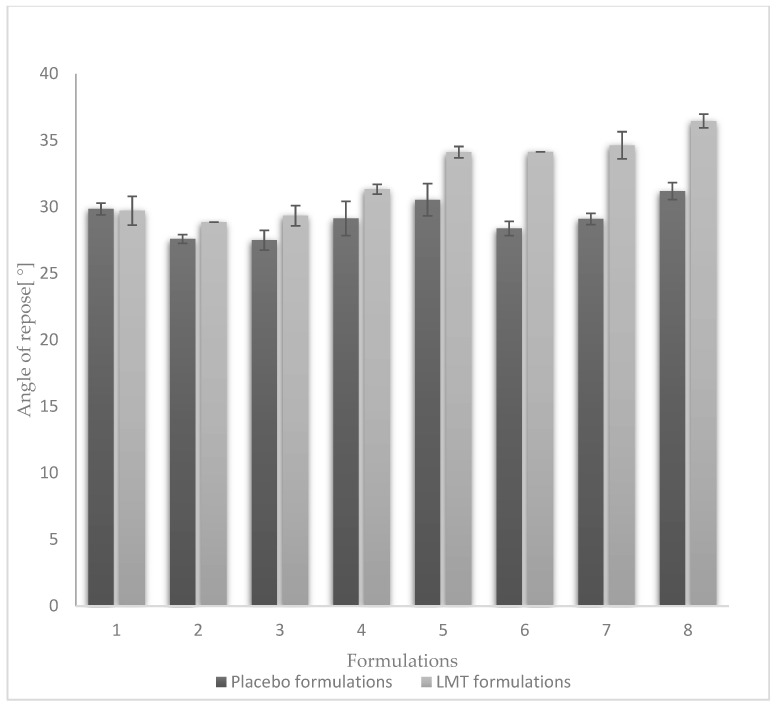
Angle of repose placebo formulations (F1–F8) and formulations with LMT (T1–T8).

A one-way analysis of ANOVA confirmed a statistically significant difference (*p* < 0.05) in angle of repose values between placebo formulations and LMT formulations when MCC was used as a filler. Angle of repose values in placebo formulations range from 26° to 36°, indicating excellent and good flowability (Ph. Eur. 10, chapter 2.9.36.) [18]. Based on the value of the angle of repose, a lower flowability was observed in formulations with the highest NaSG content and the lowest MgST content (F4, F8). Formulations with MCC F5-F8 show a smaller angle of repose compared to formulations with LAC, which is not statistically significant (*p* > 0.05). LAC has an angle of repose of 36.66°, while angles of repose of placebo formulations with LAC range from 27.49° (F3) to 29.84° (F1) because flowability is improved by the addition of MgST lubricant (Figure 2). 

In conclusion, it is observed that the addition of LMT significantly reduced the flow rate in the tableting material with MCC compared to formulations with LAC. Based on the above results, it can be noticed that all flow parameters were statistically significantly increased in formulations after the addition of LMT.

### 3.2. Characteristics of Tablets

#### 3.2.1. Resistance to Crushing of Tablets

**Figure 3:**
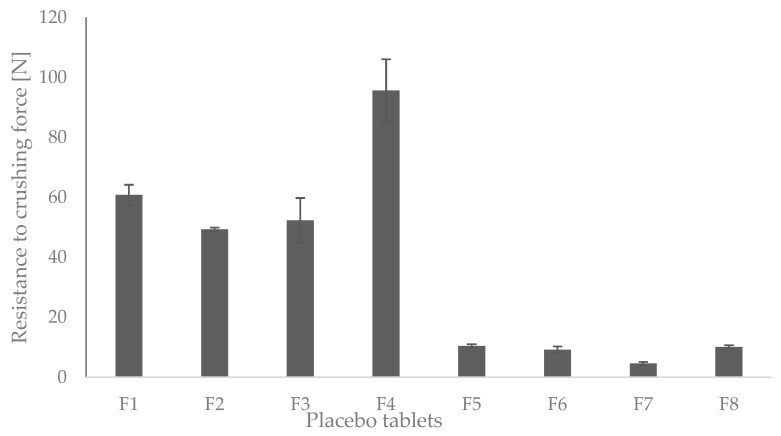
Resistance to crushing of LMT (F1–F8) and placebo tablets (T1–T8).

Same tableting conditions were used for all formulations (depth of the lower punch, height of the upper punch, i.e., the same compression force was applied), it was determined, as expected, that the resistance to crushing of the MCC formulations was statistically significantly lower, as shown in Figure 3.

The tablet formulations made under the above conditions served to partially characterize the difference between the formulations. The drawback is that the compression pressure of formulations F5–F8 was not sufficient to draw definitive conclusions. In the case of the tablet formulations that were used for further testing, a correction was made where the pressures were adjusted so that all eight formulations had a uniform breaking resistance (40–60 N).

**Figure 4:**
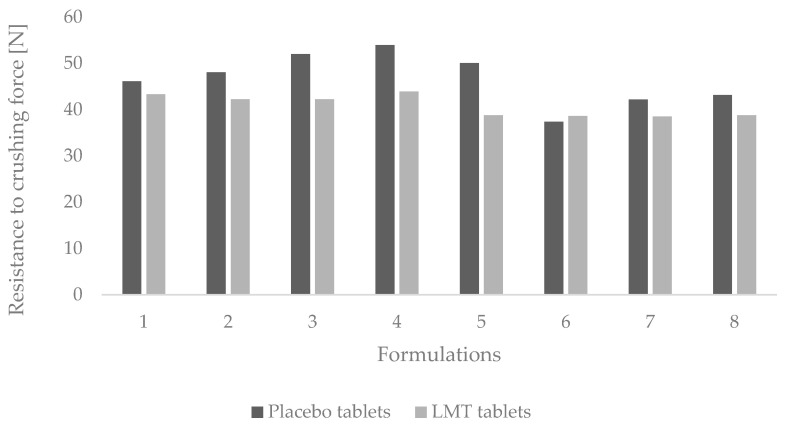
Resistance to crushing of LMT (F1–F8) and placebo tablets (T1–T8).

In tablet formulations with the addition of LMT, the T3 formulation has the highest breaking force (Figure 4). In MCC tablet formulations, fracture resistance was not significantly different between tablet formulations except for T6, which had a lower fracture resistance compared to other formulations.

#### 3.2.2. Friability

**Figure 5:**
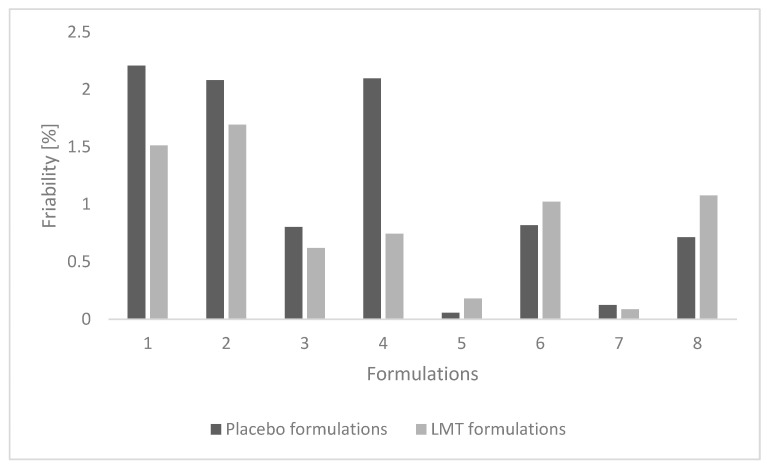
Friability of placebo (F1–F8) and LMT (T1–T8) tablet formulations.

According to the requirements of the Ph. Eur. 10, the friability of the placebo formulations was not satisfactory in the case of F1, F2, and F4 formulations (Figure 5). Percent friability was below 1% for all placebo MCC formulations, indicating that friability was within acceptable limits [18]. 

According to the requirements of the pharmacopoeia, friability was less satisfactory in tablet formulations T1 and T2 than in formulations with LAC, while in formulations with MCC in T6 and T8 it was slightly higher than 1%. In the case of other formulations, it was lower or equal to 1%, which indicates that the brittleness is within the acceptable limits required by Ph. Eur. 10 [18].

#### 3.2.3. Disintegration Time

Placebo tablet formulations with LAC have a slower disintegration time compared to MCC formulations (Figure 5). Tablets with MCC disintegrated quickly, but formulations with a higher proportion of MgST and NaSG disintegrated slower, which is also the case with formulations with LAC.

Formulations of LMT tablets with MCC had a significantly faster disintegration time than tablets with LAC. After examining the disintegration of tablets with LMT it was found that the addition of LMT to the placebo tablet leads to a faster disintegration of formulations with LAC. It is observed that formulations with a higher concentration of NaSG show shorter disintegration times compared to a formulation containing a lower proportion, especially in formulations with LAC. The higher concentration of MgST caused a prolonged disintegration time of the tablets, reducing the effect of NaSG as a superdisintegrant.

**Figure 6:**
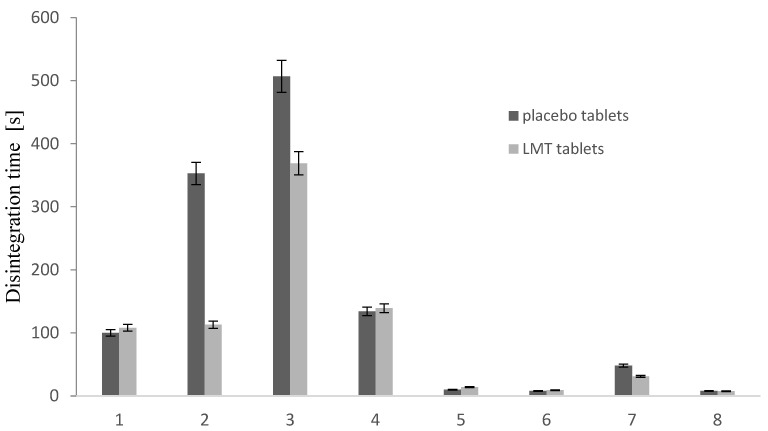
Disintegration of placebo tablet formulations and LMT tablets.

Formulations with a higher NaSG content and a lower MgST have had a shorter disintegration time. Formulation F3 with LAC as filler and F7 with MCC as filler, had the longest disintegration time compared to all other formulations. Formulations of LMT tablets with MCC had a significantly faster disintegration time than tablets with LAC. After testing the disintegration of tablets with LMT, it was found that the addition of LMT to the placebo tablet leads to a faster disintegration of formulations with LAC. From graph (Figure 6) it was observed that formulations with a higher NaSG content showed shorter degradation times compared to a formulation containing a lower content, especially in the case of LAC formulations. A higher proportion of MgST caused a prolonged tablet disintegration time, reducing the effect of NaSG as a superdisintegrator.

The formulations with the highest proportion of MgST had the longest disintegration time, as with the placebo formulation. Only the formulation with LAC F4 did not show the expected fastest disintegration time due to the higher proportion of NaSG, while the lower proportion was more efficient in the formulation with LAC. The biggest difference between the placebo tablets and the tablets after the addition of LMT was shown by the T2 formulation, where the disintegration time was reduced several times, and therefore the role of NaSG as a superdisintegrator became more pronounced. 

The higher concentration of MgST caused a prolonged disintegration time of the tablets, reducing the effect of NaSG as a superdisintegrant.

Content uniformity test results showed that all LMT formulations fit the criteria of Ph. Eur. 10 [18]. LMT formulations contain not less than 94.45% and not more than 104.5% of the labeled amount of the active drug.

#### 3.2.4. Characteristics of Tablets Stored in Conditions of Increased and Decreased Humidity

After exposing the formulations to conditions of high (75 ± 5%) and low (30 ± 5%) humidity, there was a statistically significant variation in the weight of the tablets (Table 2). Under high humidity conditions, tablets with MCC as a refilling agent had a significant increase in mass as they absorbed more moisture and this led to tablet swelling. Tablets with LAC also had an increased mass under conditions of increased moisture, but in a smaller percentage than tablets with MCC. As for LAC, the reduced moisture conditions led to a decrease in moisture, but mainly in tablets with a higher NaSG content, which had a higher moisture content in the initial conditions.

The resistance to crushing of tablets is, however, more increased in conditions of reduced humidity in formulations with LAC, which is the reason for the presence of LAC itself, because its share in tablets is the largest, which makes its impact the most pronounced. In formulations T1 and T3 (with lactose) there is an increase the resistance to crushing in conditions of reduced (30%) humidity (statistically significant), while in T2 there is no change and in T4 there was a slight decrease, but this was not statistically significant. In formulations with MCC T5, a slight increase the resistance to crushing was observed, but not statistically significant, the resistance to crushing in formulation T6 was almost unchanged. In formulations T7 and T8, there was a statistically significant decrease the resistance to crushing. 

Placebo formulations (Table 3) in which LAC was used as a supplement, in conditions of increased humidity, had a statistically significant increase in resistance to crushing, compared to tablets that were not exposed to these conditions, while in conditions of reduced humidity, they had a decrease in the measured parameter. The exception was F4, which had the highest crushing resistance after making tablets, and in conditions of high and low humidity, there was a decrease. In addition, formulations with LAC, with the exception of formulation F4, showed an increase in fracture resistance after exposure to conditions of 75% humidity.

**Figure 7:**
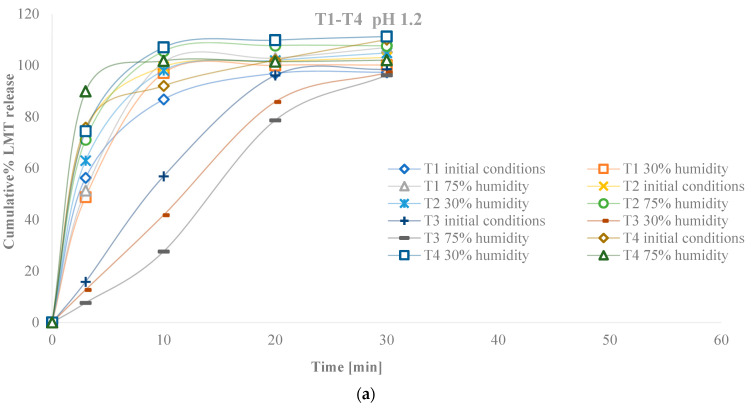

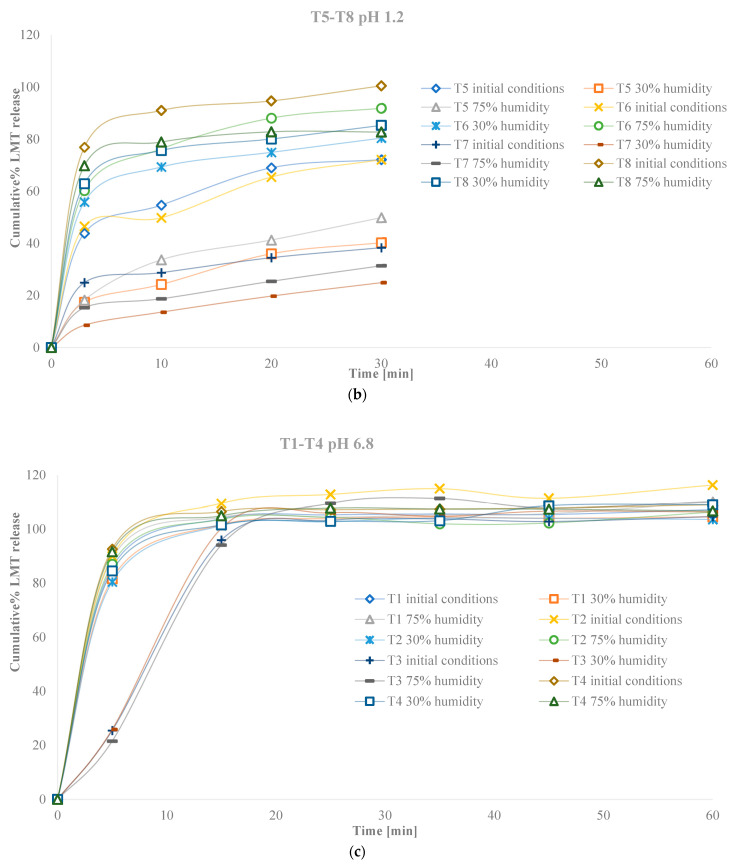

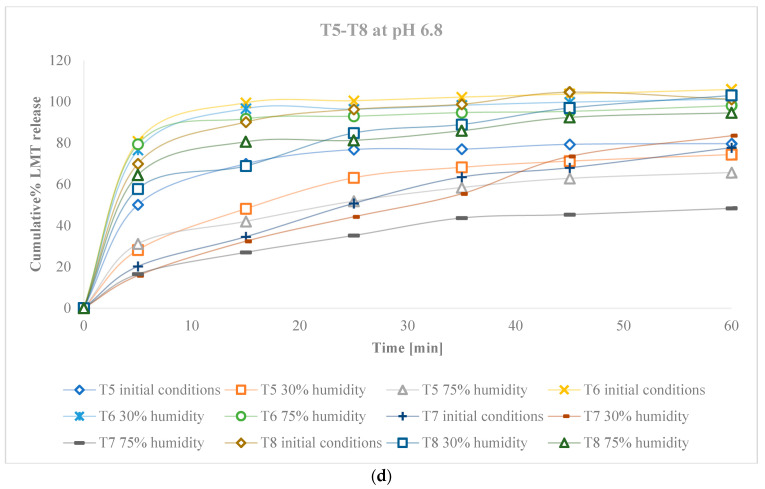
Dissolution profiles of LMT tablet formulations (**a**) T1–T4 and (**b**) T5–T8 at pH 1.2; (**c**) T1–T4 and (**d**) T5–T8 at pH 6.8 and for initial conditions, after conditions of reduced and increased humidity.

Dissolution profiles are presented in Figure 7a,b, for all humidity conditions and at both pH of dissolution medium. Formulations with LAC (T1–T4) in dissolution medium pH 6.8 showed stable release of LMT after exposure to high and low humidity conditions (Figure 7c). At pH 1.2, the slowest release of LAC formulations was shown by the formulation with the highest MgST content and the lowest NaSG (T3) content. The effect of reduced humidity on the T4 formulation led to an accelerated release of LMT. For comparison purposes separated dissolution profiles are presented in simplified dissolution profile in Suplementary Figures S1 and S2. 

Formulations with MCC (T5–T8) showed unstable release profiles after exposure to high and low humidity conditions. In the dissolution medium pH 6.8, the slowest release was shown by the T7 formulation, i.e., the formulation with the highest MgST content and the lowest NaSG content, which confirms the effect of MgST on slowing down the release of LMT. In this medium, in formulations with MCC, conditions of reduced and especially increased humidity slow down the release of LMT. In the dissolution medium pH 1.2, the slowest release of LMT, in addition to the T7 formulation, was shown by the T5 formulation after exposure to conditions, however, in the T6 formulation it is observed that exposure to high humidity significantly affects a faster LMT release.

Formulations with MCC T5 and T7 in both media, under all conditions showed incomplete release according to pharmacopeial regulations for immediate release tablets because they did not release 85% of the drug within 15 min. Formulation F8 when exposed to low and high humidity conditions had unsatisfactory release, while in the initial conditions it met the percentage of LMT released in the first 15 min.

**Figure 8:**
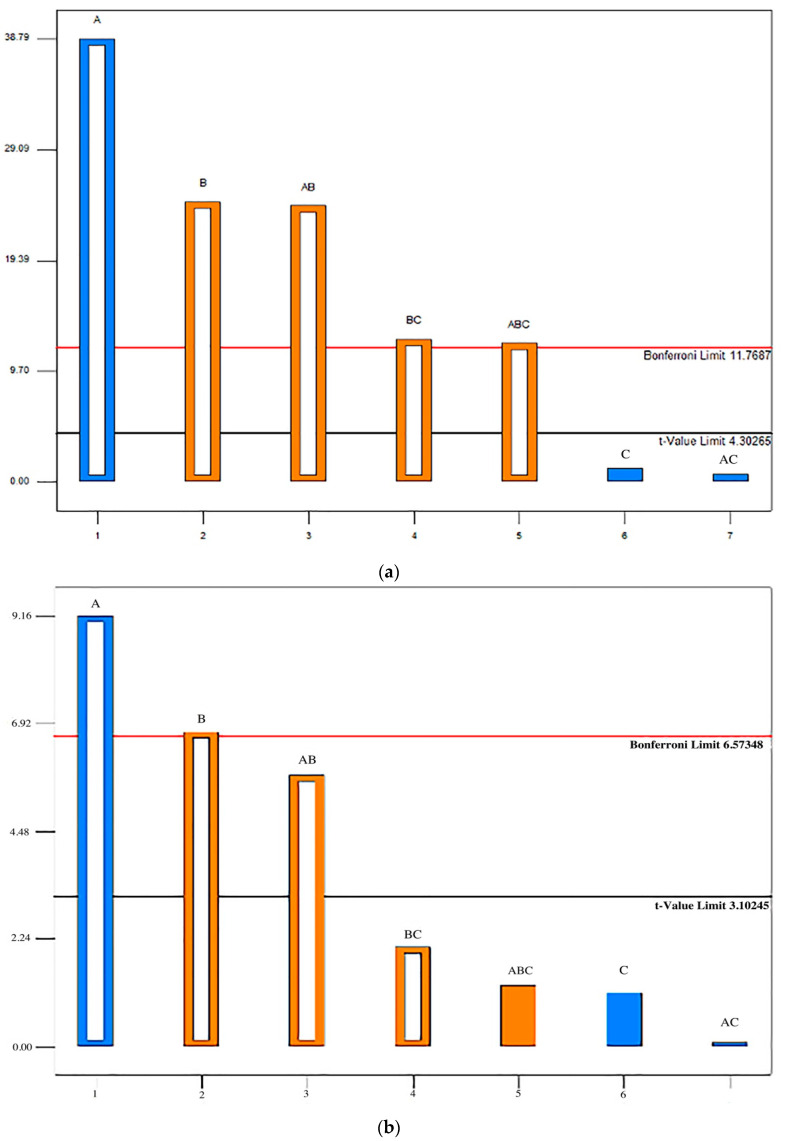

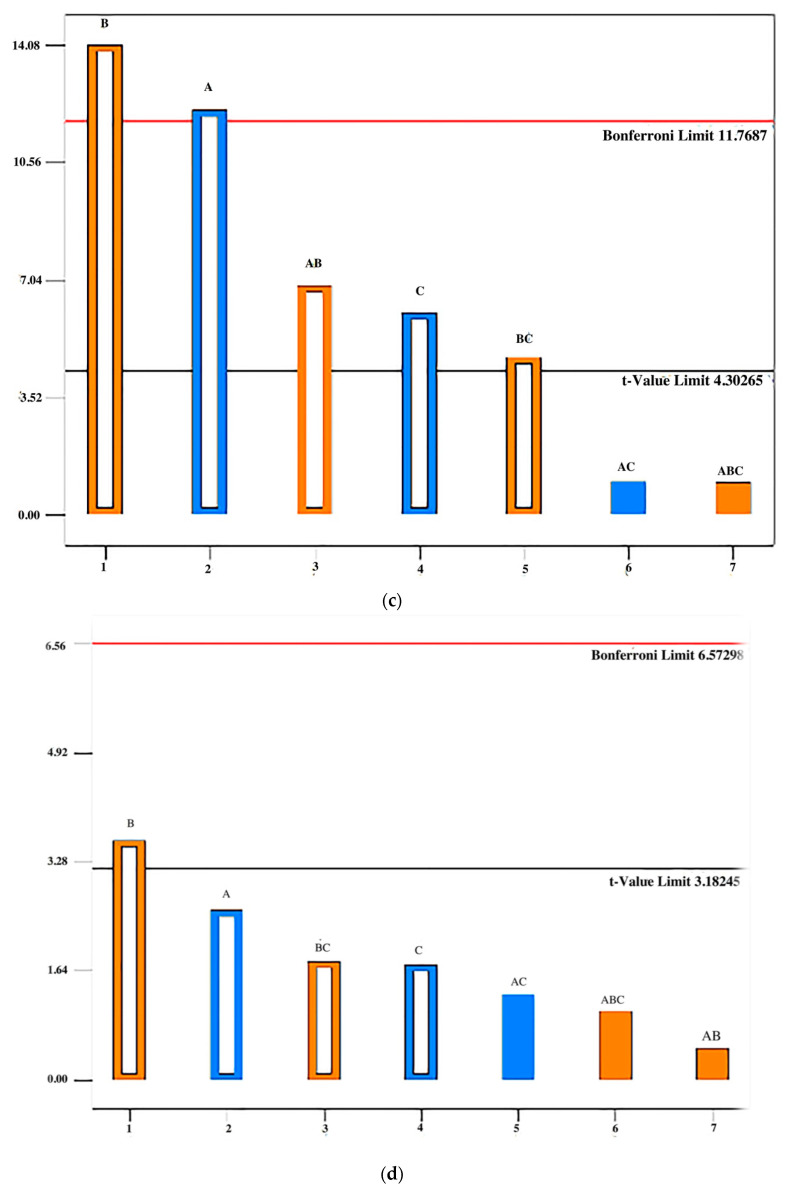
Influence of formulation components on LMT dissolution rate in 15 min in pH 6.8 dissolution medium after exposure to (**a**) high humidity conditions, (**b**) low humidity conditions; dissolution medium pH 1.2 after exposure, (**c**) high humidity conditions and (**d**) low humidity conditions. A (type of filler), B (NaSG), C (MgST).

Based on the Pareto diagram (Figure 8), the type of filler (factor A) showed the greatest influence on the dissolution of API in the dissolution medium pH 6.8 under conditions of low and high humidity. Bonferroni limit is the threshold above which the effects that emerge are significant (very important). In this case, the concentration of NaSG (factor B), as well as its interaction with MCC and LAC (factor AB), showed statistical significance that was above the Bonferroni limit. The effect terms below the threshold of the t-limit are insignificant factors The interaction of MgST and NaSG (factor BC), as well as the interaction of all three excipients (factor ABC), showed statistical significance in low humidity conditions (30%) and were between Bonferroni and t-value limit.

In the pH dissolution medium 1.2, the disintegrant showed the greatest influence on the release of LMT, which showed the only statistical significance when exposed to high humidity, while in low humidity conditions the choice of excipients, such as MgST and their interactions (Figure 8).

## 4. Discussion

The flow of tableting material is a critical factor during compression because it affects the uniformity of the filling, as well as the mechanical characteristics of the manufactured tablets, which in turn affects the dissolution rate of the compound. This is especially important with poorly soluble compounds, such as LMT.

The effect of humidity in a short period of four weeks was examined in order to determine the extent to which humidity affects the LMT release profile if the tablet is exposed to these conditions, which is realistically possible if the drug is separated from the original packaging, which could occur in preparation for weekly or monthly therapy. Exposure of LMT tablet formulations to conditions of high humidity led to changes in the characteristics of the tablets and reduced the rate of LMT release. Thus, after repackaged in pill organizers, the phase of formulation development conditions should be taken into consideration. 

Previous literature data confirm that capillary bridges are formed between LAC particles in conditions under high humidity, and thus cohesion interaction is enhanced. It has been confirmed that storage of LAC in conditions of elevated humidity above 65%, capillary force and Lifshitz-van der Waltz force usually dominate over interparticle force, while in conditions below 65% interparticle force mainly dominates over Lifshitz–van der Waals force and electrostatic force [21]. The results showed an improvement in the flowability of powders in storage conditions to 75% humidity, which occurs in formulations with MCC and a higher concentration of MgST, and a lower concentration of NaSG. Many authors have suggested that the humidity content of MgST formulations may significantly affect the physicochemical characteristics of the pharmaceutical form [22,23]. Despite the hydrophobic nature of MgST, this study found that some physical characteristics of MgST change under conditions of high humidity and that the addition of water resulted in a better lubricating effect of MgST.

In MCC formulations, the increased humidity content had a negative effect on the breakage resistance of the tablets. Other studies have obtained similar data. Concluded that increased humidity (75%,) leads to softening of tablets and swelling in formulations with MCC [22]. Humidity inside the pores of the MCC can act as an internal lubricant, reduces friction and facilitates sliding, allows better transmission of compression force through the compact and reduces the adhesion of tablets to the matrix of the tablet. Pilpel and Ingham studied the effect of humidity in MCC on density, porosity, and tensile strength [24]. They linked changes in MCC mechanical properties and tensile strength of compact materials to the way water is absorbed into the cellulosic structure by binding one water molecule between two anhydroglucose units, followed by binding one water molecule to each anhydroglucose unit, increasing MCC molecular mobility and may explain why water acts as a plasticizer.

Conditions of increased and decreased humidity led to an increase in resistance to crushing of LMT formulations, which is different from placebo formulations. This can be explained by the already assumed interpretation based on the previous results that LMT also acts as a lubricant, whereby with an increase in moisture, due to its pronounced hydrophobicity, it leads to an enhanced lubricant effect for which certain studies have already shown that in conditions of increased moisture, they can increase the resistance to breaking into tablets [25].

LAC tablets with a higher concentration of MgST (F2 and F3) after four weeks led to a decrease of resistance to crushing. This is explained by the fact that atmospheric water is absorbed by a predominantly hygroscopic amorphous lactose fraction, and the initial increase in resistance to breakage is considered to be lubrication with water. However, the water content affects the transition of amorphous lactose from the glassy state to the rubber state, resulting in mutarotation and crystallization, and a decrease in the compressibility of the excipient has been demonstrated [23]. Decreased humidity mainly affected the increase of resistance to crushing in formulations with LAC, especially in formulations with increased NaSG content in which the humidity was reduced the most after exposure to conditions of 30% humidity.

In formulations with increased MgST content, when exposed to different humidity conditions, there was a significant difference in LMT degradation and release. Therefore, determining the effect of MgST on flow rate as well as on tablet disintegration, especially in high and low humidity conditions, significantly contributes to the formulation of an optimal tablet with a precisely determined release time of pharmaceutically active substances. The possible reason for the deviation of formulations with a high content of NaSG and MgST due to the confirmed influence of the interaction of these two excipients has already been considered.

Regarding the LMT release profile from tablets exposed to these conditions, LAC formulations (T1–T4) in a stable pH 6.8 dissolution medium showed stable LMT release after exposure to high and low humidity conditions. There was a statistically significant difference in the dissolution medium pH 1.2, the slowest release in LAC formulations was shown by the formulation with the highest MgST content and the lowest NaSG content (T3) and confirmed interaction. The effect of reduced humidity on the T4 formulation led to an accelerated release of LMT, which was most likely influenced by the high NaSG content. 

In contrast to LAC formulations, MCC formulations showed unstable API release profiles after exposure to different humidity conditions, confirming the significant effect of storage conditions on formulations using MCC as an additive. At pH 6.8 in MCC formulations, conditions of reduced and especially elevated humidity slowed the release of LMT. In the pH 6.8 dissolution medium, the slowest release was shown by the T7 formulation, i.e., the formulation with the highest MgST content and the lowest NaSG content, which confirms the effect of MgST on the slowing down of API release.

Up to date, studies have confirmed the effect of MgST on release rates in weak bases [26,27,28,29]. MgST can change the pH of the microenvironment and slow the release of LMT. In pH 1.2 dissolution medium, the slowest release with the T7 formulation was shown by the T5 formulation after exposure to high and low humidity conditions, however, in the T6 formulation it was observed that exposure to high humidity conditions significantly affects a faster LMT release. Formulations with MCC T5 and T7 in both media, under all humidity conditions, showed incomplete release according to pharmacopeial regulations for instant-release tablets because they did not release 85% of the drug within 15 min. The reason for the lower release of T5 and T7 formulations is the low NaSG content as this is a common feature for both formulations. Regardless of the stability of LMT, the cause of instability may be excipients that cause certain changes in the finished pharmaceutical form. The development of new delivery systems, such as nano formulations [30,31,32], or improved solid dosage formulation with adjusted drug release [33,34,35] has been in the current focus of many recent researches where robust stability was proven when exposed to different temperatures and/humidity conditions. However solid dosage forms represent major market share, where tablets with immediate drug release are holding are still dominantly produced and marketed all over the world. Thus, more profound knowledge in the formulations design, stability and excipients interaction in is very valuable in order to produce product that is stable and safe for the use. Examining the behavior of a formulation with different ratios of excipients under the influence of external factors such as different temperature and humidity during storage significantly contributes to the detection of different interactions between the components of the mixture. The excipients themselves may be affected by chemical, physical, or microbiological instability. Physical instability includes the phase transformation of auxiliary materials, which may be caused by polymorphic changes, hydration and dehydration, deposition, or changes of an amorphous or crystalline nature. In this study, common excipients were examined in tablet formulations. The data of examined placebo and LMT tablets prepared using common excipients that are frequent in LMT formulations pointed out that both composition of formulation and interactions of excipients are relevant to stability of characteristics, such as disintegration time and dissolution after exposure to different humidity conditions.

## 5. Conclusions

After four weeks of exposure of the tablet material to conditions of increased humidity, it was observed that in formulations with LAC, MgST improves flowability, however it decreases in formulations with MCC. 

It was found that different proportions of excipients influence the rate of release of LMT from tablets under the influence of increased and decreased humidity. As an excipient, lactose showed more stable formulations with respect to the effect of moisture on API release.

Conditions of increased and decreased humidity did not significantly affect the release of LMT from formulations T2 and T4 (formulations with a higher NaSG content), while there was reduced release of LMT with formulations T1 and T3 (formulations with a lower NaSG content).

Placebo formulation F1–F4 with LAC at a lower humidity increased resistance to crushing, and formulations F5–F8 with MCC as filler were not affected. However, at a high humidity placebo formulations F1–F4 were less affected than formulations F5–F8, where decrease in resistance to crushing was detected.

Confirmed changes in tablet characteristics, particularly in release rate after exposure to different humidity conditions, indicates significance of excipient selection during formulation development and impact of repackaging of drugs that should be addressed more closely, especially in guidelines for safekeeping and use of drug formulations.

## Figures and Tables

**Table 1:** Composition of immediate release LMT tablet formulations.

Formulations ***	LAC	MCC	MgST [%]	NaSG [%]	LMT [%] Adjusted for Dose 25 mg per Tablet
T1	q.s.*	/	0.25	0.5	q.s. **
T2	q.s.	/	5	4	q.s. **
T3	q.s.	/	5	0.5	q.s. **
T4	q.s.	/	0.25	4	q.s. **
T5	/	q.s.	0.25	0.5	q.s. **
T6	/	q.s.	5	4	q.s. **
T7	/	q.s.	5	0.5	q.s. **
T8	/	q.s.	0.25	4	q.s. **

* q.s. lat. quantum satis or as needed, ** LMT dose in each tablet was 25 mg, and % depends on tablet mass reached on having resistance to crushing of 60–90 N, *** dependent variables were angle of repose, Hausner ratio, Carr’s index, resistance to crushing, weight variation, moisture content, dissolved amount after 15 and 30 min at pH 1.2 and pH 6.8.

**Table 2:** Test values for moisture content in LMT tablet formulations (T1–T8), resistance to crushing and weight variation (results are shown as mean value ± standard deviation.

Conditions	Formulations	Moisture Content [%]	Crushing Force [N]	Weight Variation [g]	LMT Content [%]
Initials	T1	0.16 ± 0.04	53.48 ± 2.11	0.56 ± 0.01	97.41 ± 8.55
	T2	0.72 ± 0.06	56.35 ± 9.38	0.54 ± 0.02	94.44 ± 3.13
	T3	0.27 ± 0.08	50.225 ± 4.69	0.54 ± 0.02	95.27 ± 5.20
	T4	0.49 ± 0.09	53.90 ± 8.00	0.57 ± 0.02	97.63 ± 8.89
	T5	3.63 ± 0.49	56.35 ± 2.68	0.34 ± 0.01	100.48 ± 1.97
	T6	4.59 ± 0.31	62.88 ± 6.51	0.36 ± 0.02	99.12 ± 6.22
	T7	3.88 ± 0.52	74.725 ± 2.45	0.34 ± 0.02	103.90 ± 11.89
	T8	4.59 ± 0.31	58.80 ± 6.93	0.34 ± 0.02	99.89 ± 4.98
30% humidity	T1	0.12 ± 0.03	69.83 ± 4.69	0.55 ± 0.01	93.55 ± 3.18
	T2	0.52 ± 0.01	52.68 ± 2.45	0.54 ± 0.01	96.17 ± 5.52
	T3	0.19 ± 0.10	72.77 ± 4.26	0.54 ± 0.01	101.22 ± 3.87
	T4	0.43 ± 0.07	44.10 ± 12.00	0.53 ± 0.01	99.99 ± 4.89
	T5	3.06 ± 0.27	61.25 ± 4.90	0.34 ± 0.01	95.17 ± 3.69
	T6	3.69 ± 0.55	61.99 ± 2.32	0.35 ± 0.01	103.22 ± 2.54
	T7	3.11 ± 0.97	68.60 ± 4.00	0.35 ± 0.01	101.29 ± 9.89
	T8	3.98 ± 0.28	49.82 ± 4.82	0.33 ± 0.01	97.93 ± 3.75
75% humidity	T1	0.47 ± 0.22	99.63 ± 12.65	0.55 ± 0.01	98.18 ± 7.81
	T2	1.81 ± 0.23	64.19 ± 0.98	0.55 ± 0.01	92.25 ± 4.11
	T3	1.14 ± 0.00	88.20 ± 5.66	0.54 ± 0.01	98.20 ± 4.80
	T4	1.52 ± 0.07	45.33 ± 6.17	0.55 ± 0.01	95.23 ± 9.03
	T5	7.01 ± 0.10	66.15 ± 2.83	0.35 ± 0.01	99.49 ± 0.55
	T6	7.07 ± 0.02	27.83 ± 4.68	0.37 ± 0.01	100.98 ± 4.35
	T7	6.50 ± 0.05	46.06 ± 2.68	0.35 ± 0.01	104.22 ± 6.27
	T8	6.84 ± 0.10	27.44 ± 2.68	0.34 ± 0.01	98.12 ± 3.37

**Table 3:** Test values for moisture content in placebo (F1–F8) resistance to crushing and weight variation (results are shown as mean value ± standard deviation.

Conditions	Formulations	Moisture Content [%]	Crushing Force [N]	Weight Variation [g]
Initials	F1	0.26 ± 0.07	57.21 ± 4.82	0.55 ± 0.01
	F2	0.75 ± 0.03	62.11 ± 5.62	0.55 ± 0.02
	F3	0.37 ± 0.03	53.94 ± 4.77	0.56 ± 0.01
	F4	0.96 ± 0.07	96.43 ± 8.35	0.56 ± 0.02
	F5	4.33 ± 0.42	60.46 ± 4.82	0.35 ± 0.01
	F6	4.69 ± 0.31	59.81 ± 7.33	0.36 ± 0.02
	F7	3.98 ± 0.32	54.58 ± 3.52	0.34 ± 0.02
	F8	4.29 ± 0.51	59.81 ± 4.77	0.35 ± 0.02
30% humidity	F1	0.32 ± 0.01	48.71 ± 4.32	0.55 ± 0.01
	F2	0.71 ± 0.02	84.99 ± 7.52	0.55 ± 0.02
	F3	0.30 ± 0.07	65.70 ± 8.85	0.53 ± 0.01
	F4	0.51 ± 0.11	106.24 ± 9.29	0.53 ± 0.01
	F5	3.16 ± 0.31	61.44 ± 6.12	0.35 ± 0.03
	F6	3.88 ± 0.45	60.13 ± 5.21	0.34 ± 0.01
	F7	3.54 ± 0.89	59.81 ± 5.11	0.35 ± 0.01
	F8	3.92 ± 0.31	62.75 ± 4.33	0.34 ± 0.01
75% humidity	F1	1.41 ± 0.22	67.01 ± 6.12	0.56 ± 0.01
	F2	2.04 ± 0.23	71.92 ± 5.28	0.52 ± 0.02
	F3	2.63 ± 0.00	70.28 ± 6.34	0.54 ± 0.01
	F4	1.82 ± 0.47	39.23 ± 2.88	0.55 ± 0.02
	F5	7.96 ± 0.14	53.13 ± 3.88	0.37 ± 0.01
	F6	7.96 ± 0.13	49.81 ± 3.23	0.36 ± 0.01
	F7	6.71 ± 0.21	48.50 ± 3.22	0.36 ± 0.01
	F8	6.74 ± 0.32	50.46 ± 2.87	0.35 ± 0.01

## Data Availability

Not applicable.

## Supplementary Materials

The following supporting information can be downloaded at: https://www.mdpi.com/article/10.3390/pharmaceutics14102096/s1, Figure S1: Dissolution profiles of LMT tablet formulations at dissolution medium pH 1.2 before and after exposure to conditions of reduced (30%) and increased (75%) humidity for formulation T1–T8 (a–h). Figure S2: Dissolution profiles of LMT tablet formulations at dissolution medium pH 6.8 before and after exposure to conditions of reduced (30%) and increased (75%) humidity for formulation T1–T8 (a–h).
