# Supplementary material for: The Effect of Humidity on the Dissolution Kinetics and Tablet Properties of Immediate-Release Tablet Formulation Containing Lamotrigine
Source: Pharmaceutics. 2022 Sep 30;14(10):2096. doi: 10.3390/pharmaceutics14102096 (PMC9608154; doi:10.3390/pharmaceutics14102096)
Supplement: Supplementary file 1 [file pharmaceutics-14-02096-s001.zip › pharmaceutics-1911467-supplementary.pdf]

pharmaceutics

MDPI

Supplementary Materials: The Effect of Humidity on the Dissolution Kinetics and Tablet Properties of Immediate-Release Tablet Formulation Containing Lamotrigine

Mladena Lalić-Popović, Gordana Švonja Parezanović, Nemanja Todorović, Zoran Zeković, Branimir Pavlić, Nataša Milošević, Jelena Čanji Panić, Ana Stjepanović and Ljiljana Andrijević

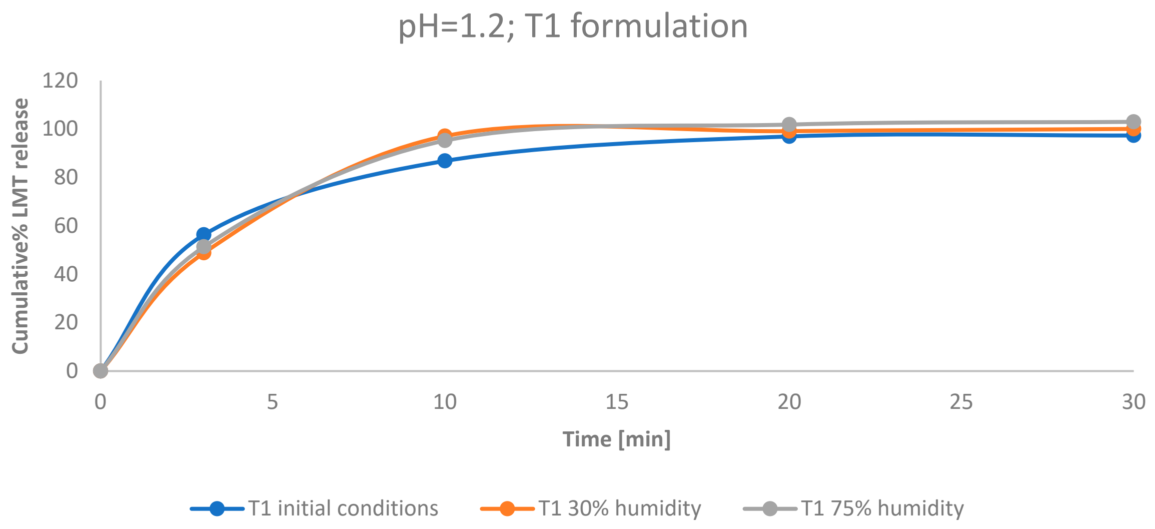

(a)

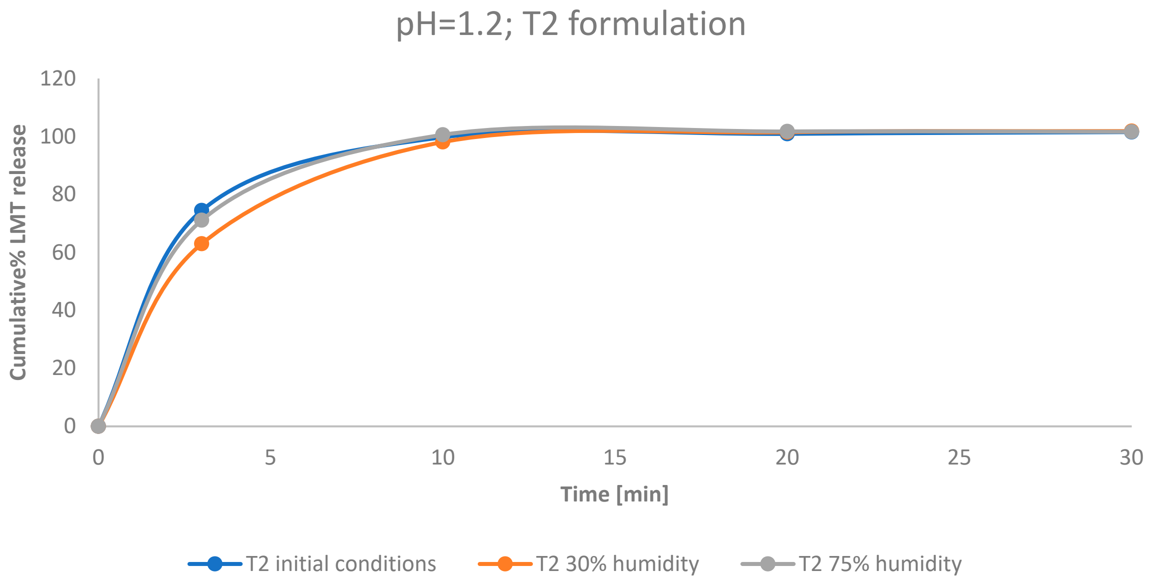

(b)

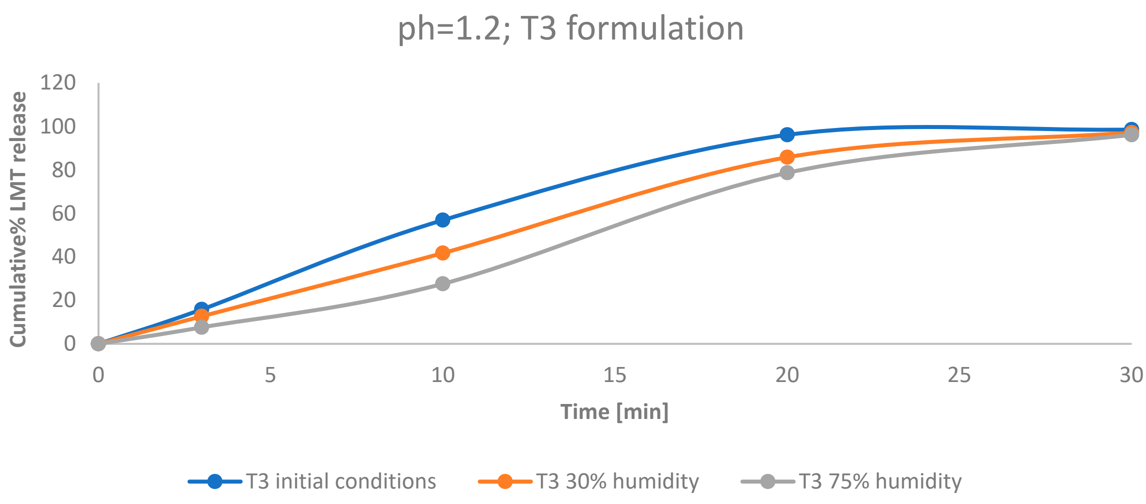
ph=1.2; T3 formulation
Cumulative% LMT release
120
100
80
60
40
20
0
0
5
10
15
20
25
30
Time [min]
T3 initial conditions
T3 30% humidity
T3 75% humidity

(c)

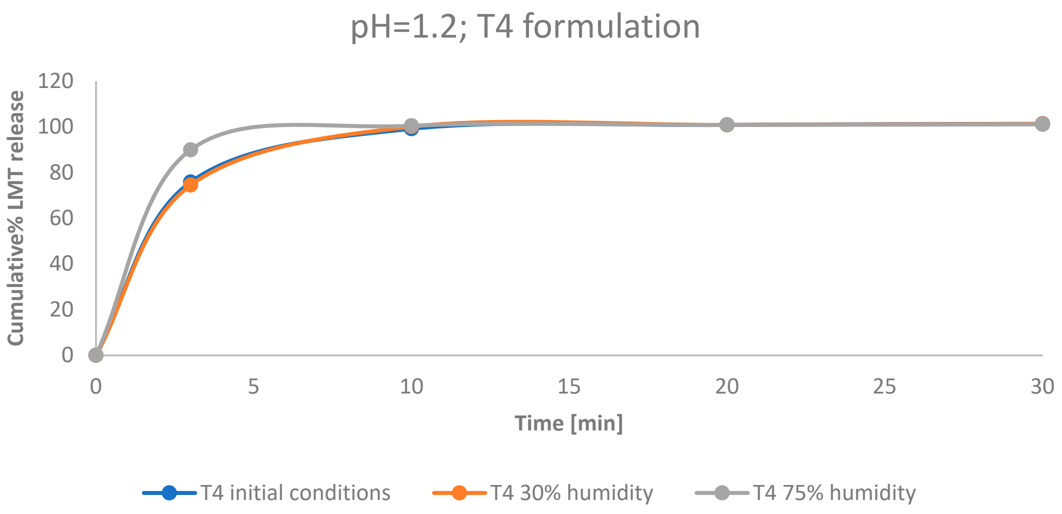
pH=1.2; T4 formulation
Cumulative% LMT release
120
100
80
60
40
20
0
0
5
10
15
20
25
30
Time [min]
T4 initial conditions
T4 30% humidity
T4 75% humidity

(d)

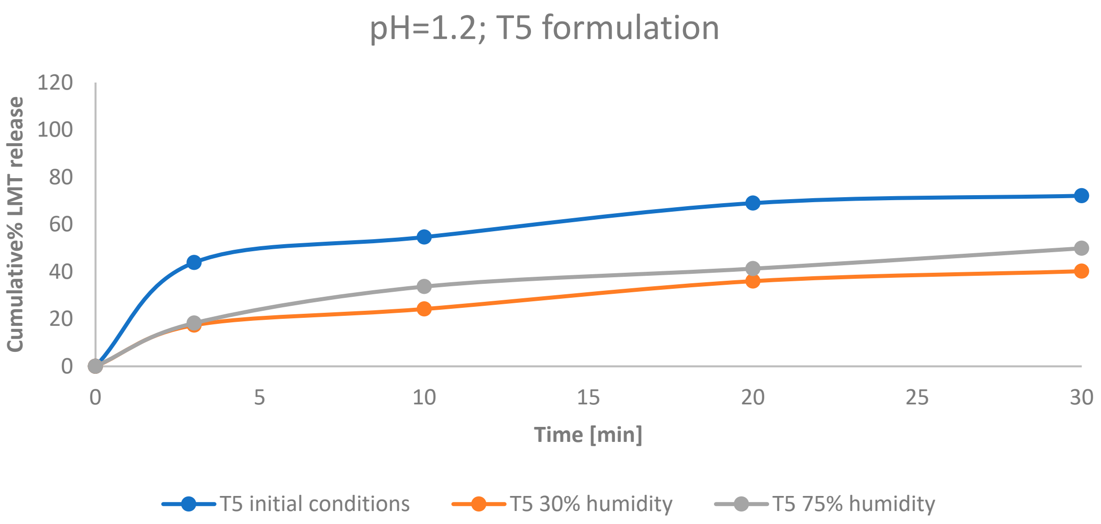
pH=1.2; T5 formulation
Cumulative% LMT release
120
100
80
60
40
20
0
0
5
10
15
20
25
30
Time [min]
T5 initial conditions
T5 30% humidity
T5 75% humidity

(e)

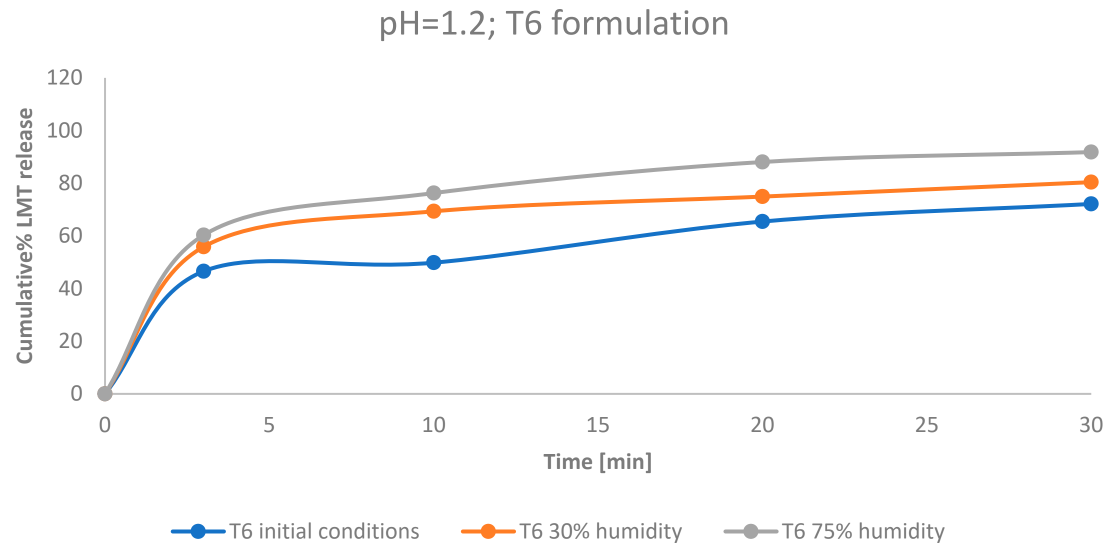

(f)

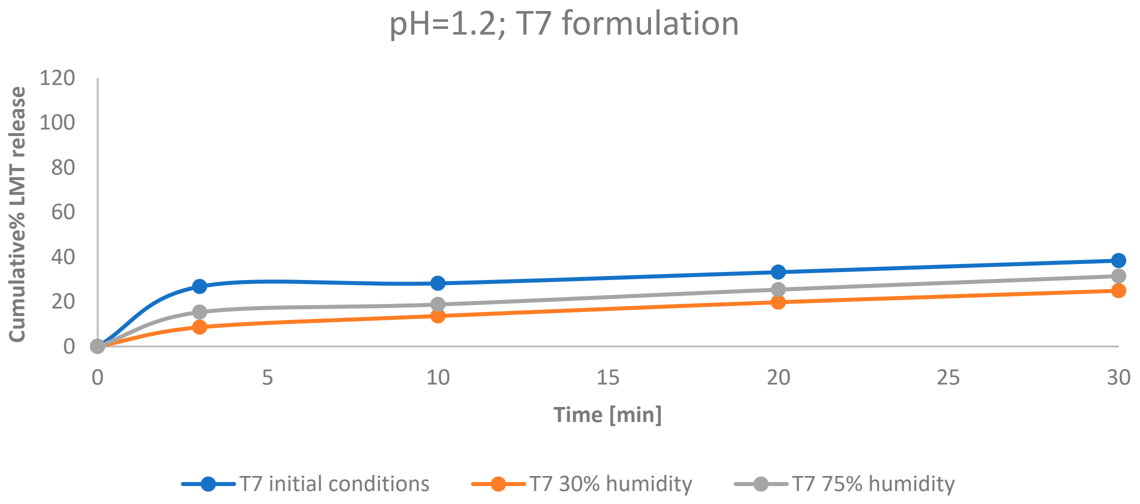

(g)

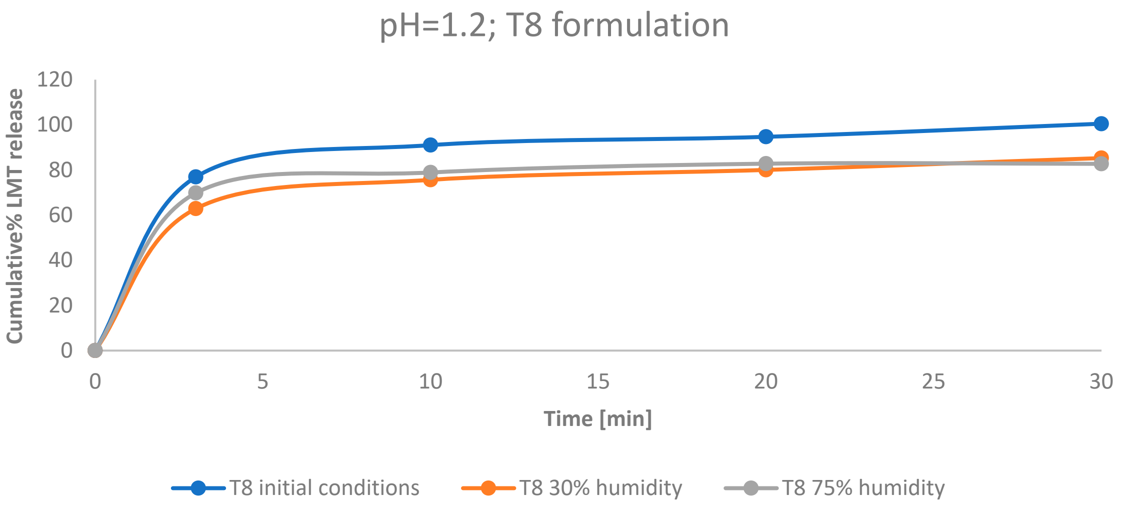

(h)

Figure S1. Dissolution profiles of LMT tablet formulations at dissolution medium pH 1.2 before and after exposure to conditions of reduced (30%) and increased (75%) humidity for formulation T1–T8 (**a**–**h**).

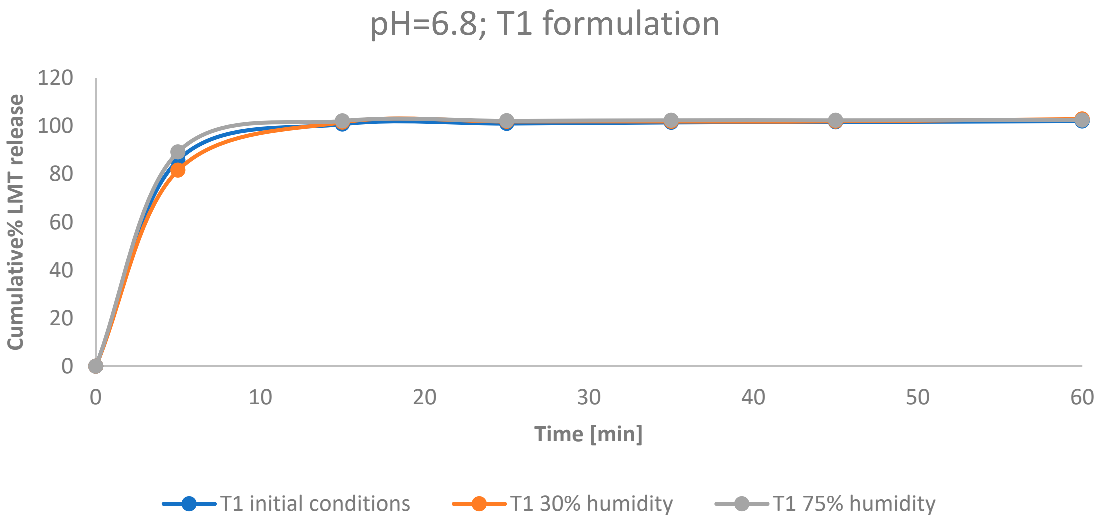
pH=6.8; T1 formulation
Cumulative% LMT release
120
100
80
60
40
20
0
0
10
20
30
40
50
60
Time [min]
T1 initial conditions
T1 30% humidity
T1 75% humidity

(a)

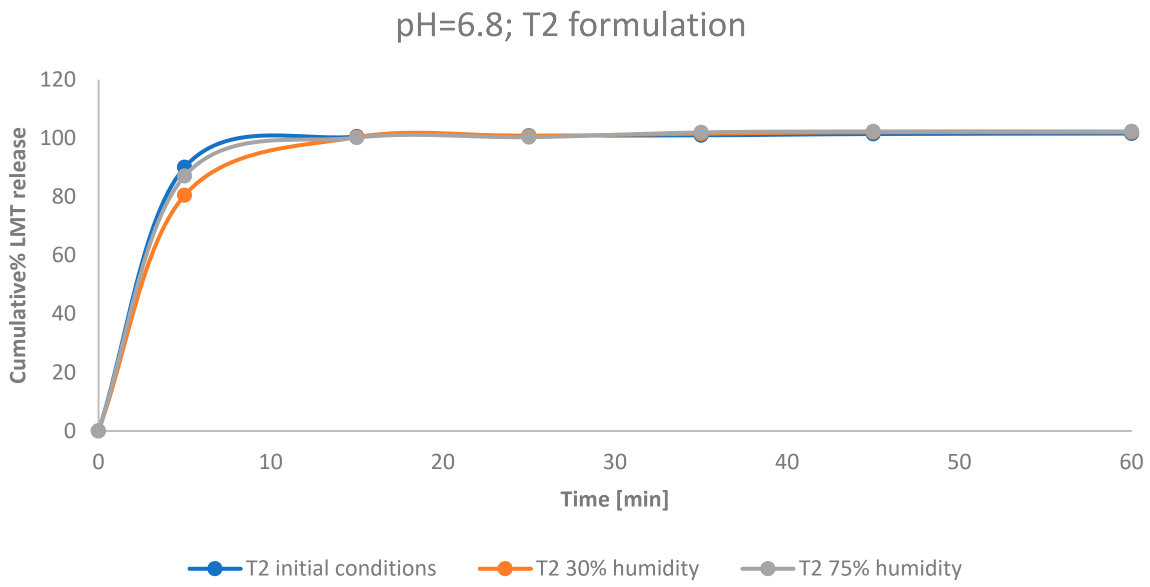
pH=6.8; T2 formulation
Cumulative% LMT release
120
100
80
60
40
20
0
0
10
20
30
40
50
60
Time [min]
T2 initial conditions
T2 30% humidity
T2 75% humidity

(b)

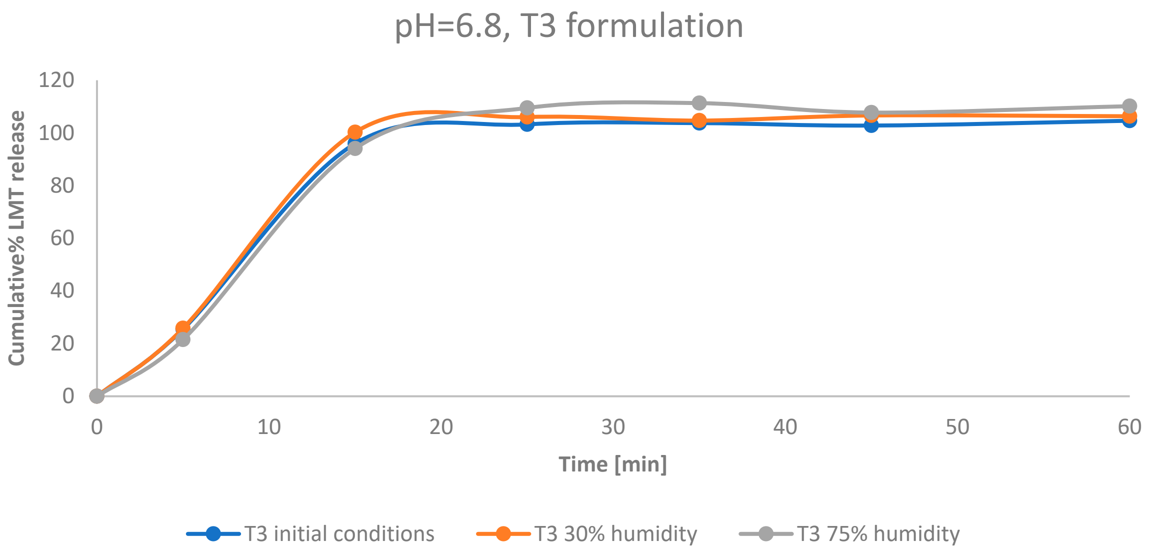
pH=6.8, T3 formulation
Cumulative% LMT release
120
100
80
60
40
20
0
0
10
20
30
40
50
60
Time [min]
T3 initial conditions
T3 30% humidity
T3 75% humidity

(c)

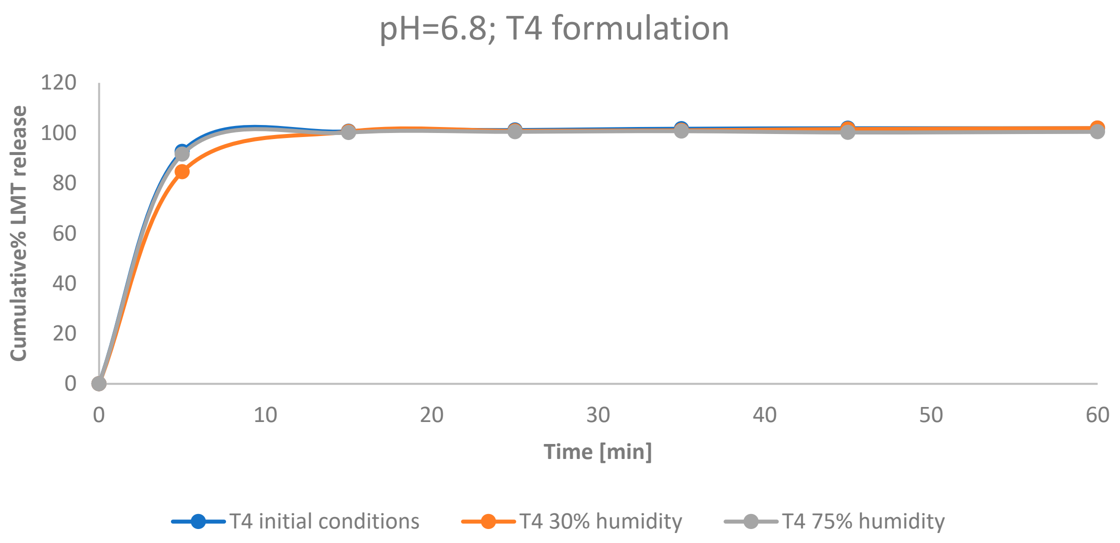
pH=6.8; T4 formulation
Cumulative% LMT release
Time [min]
T4 initial conditions
T4 30% humidity
T4 75% humidity

(d)

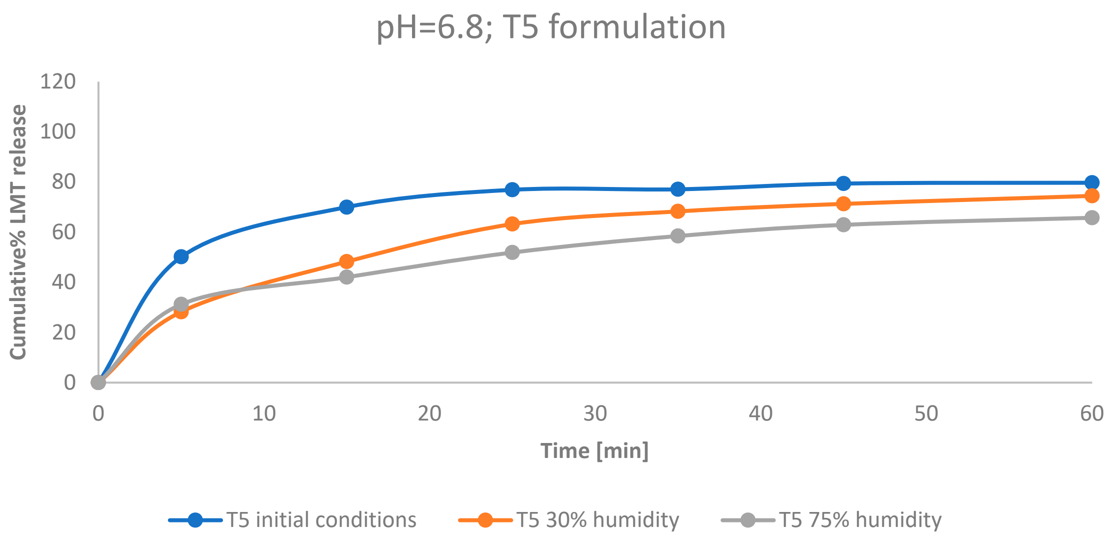
pH=6.8; T5 formulation
Cumulative% LMT release
Time [min]
T5 initial conditions
T5 30% humidity
T5 75% humidity

(e)

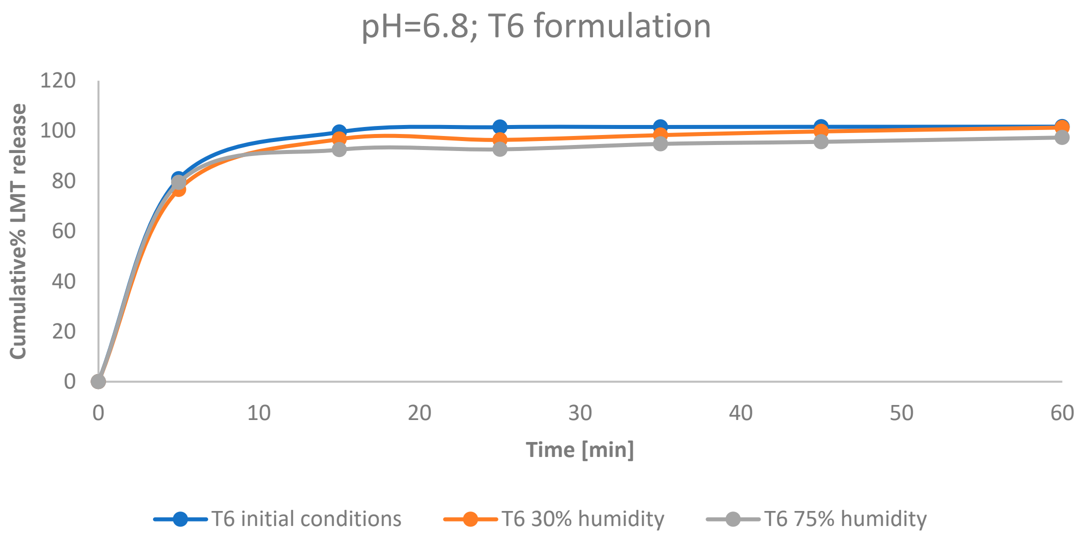
pH=6.8; T6 formulation
Cumulative% LMT release
Time [min]
T6 initial conditions
T6 30% humidity
T6 75% humidity

(f)

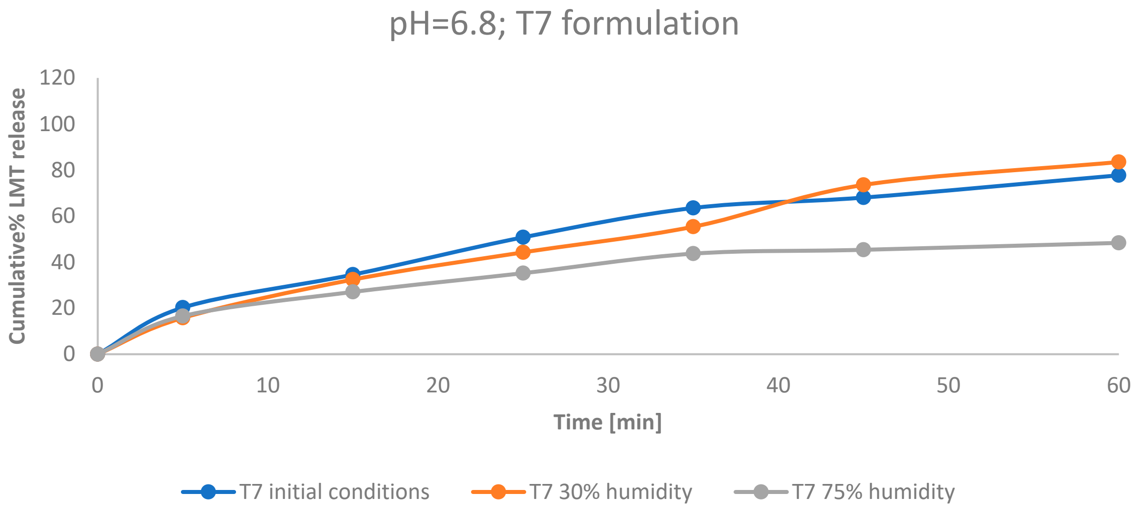

(g)

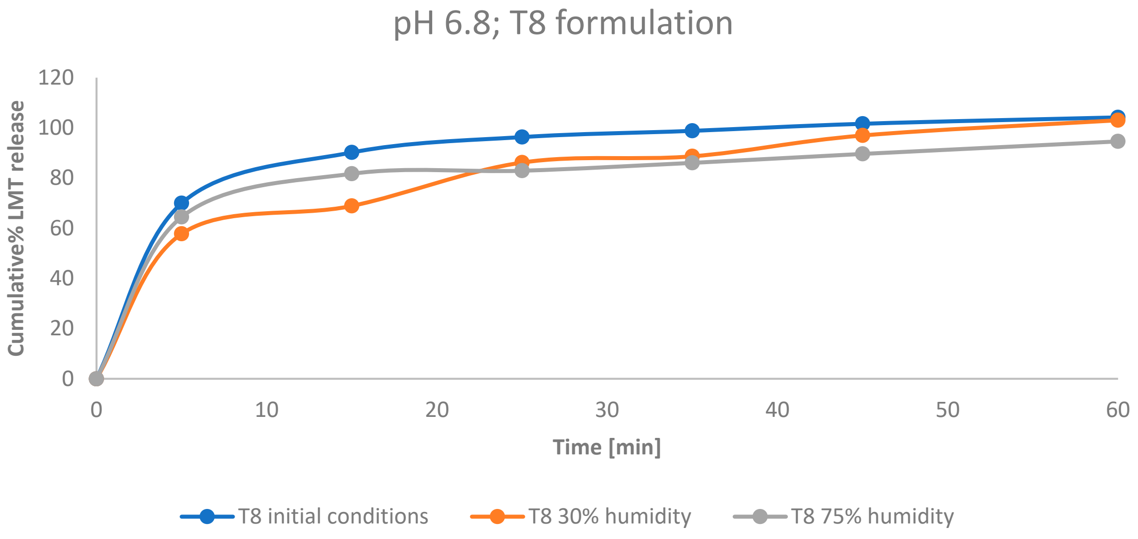

(h)

Figure S2. Dissolution profiles of LMT tablet formulations at dissolution medium pH 6.8 before and after exposure to conditions of reduced (30%) and increased (75%) humidity for formulation T1–T8 (**a**–**h**).
